# Welding Seam Tracking and Inspection Robot Based on Improved YOLOv8s-Seg Model

**DOI:** 10.3390/s24144690

**Published:** 2024-07-19

**Authors:** Minghu Zhao, Xinru Liu, Kaihang Wang, Zishen Liu, Qi Dong, Pengfei Wang, Yaoheng Su

**Affiliations:** 1 School of Science, Xi’an Polytechnic University, Xi’an 710048, China; 220811074@stu.xpu.edu.cn (M.Z.); 42208020112@stu.xpu.edu.cn (K.W.); 42208210125@stu.xpu.edu.cn (Z.L.); 42208020229@stu.xpu.edu.cn (Q.D.); 2 School of Electronic Information, Xi’an Polytechnic University, Xi’an 710048, China; 42103040131@stu.xpu.edu.cn; 3 School of Mechanical and Electrical Engineering, Xi’an Polytechnic University, Xi’an 710048, China; 42102310210@stu.xpu.edu.cn

**Keywords:** seam tracking, YOLOv8s-seg, lightweight design, weld identification, detection robot

## Abstract

A weld is the main connection form of special equipment, and a weld is also the most vulnerable part of special equipment. Therefore, an effective detection of a weld is of great significance to improve the safety of special equipment. The traditional inspection method is not only time-consuming and labor-intensive, but also expensive. The welding seam tracking and inspection robot can greatly improve the inspection efficiency and save on inspection costs. Therefore, this paper proposes a welding seam tracking and inspection robot based on YOLOv8s-seg. Firstly, the MobileNetV3 lightweight backbone network is used to replace the backbone part of YOLOv8s-seg to reduce the model parameters. Secondly, we reconstruct C2f and prune the number of output channels of the new building module C2fGhost. Finally, in order to make up for the precision loss caused by the lightweight model, we add an EMA attention mechanism after each detection layer in the neck part of the model. The experimental results show that the accuracy of weld recognition reaches 97.8%, and the model size is only 4.88 MB. The improved model is embedded in Jetson nano, a robot control system for seam tracking and detection, and TensorRT is used to accelerate the reasoning of the model. The total reasoning time from image segmentation to path fitting is only 54 ms, which meets the real-time requirements of the robot for seam tracking and detection, and realizes the path planning of the robot for inspecting the seam efficiently and accurately.

## 1. Introduction

With the continuous advancement of the industrialization process, the application of steel plates in the production and manufacturing of equipment is becoming more and more extensive. These steel plates are connected by welds to build large-scale equipment similar to hull decks [1], steel structure bridge decks [2], and other horizontal arrangements. They play an important role in many industries such as energy [3], navigation [4], and transportation [5]. However, there are many challenges related to the quality of steel plate weld joints. For example, due to factors such as a harsh application environment and bearing gravity, common problems and defects such as corrosion, surface peeling, cracks, and fatigue fracture may occur at steel plate weld joints [6]. The existence of these defects and damages undoubtedly poses a potential threat to the safe use of steel plates, and is prone to dangerous situations such as hull deck collapse [7] and steel bridge fracture [8], which pose a threat to human life and safety. Traditional weld inspection mainly relies on manual measurement and empirical judgment [9]. However, this completely manual method is not only time-consuming, labor-intensive, and inefficient, but also dangerous [10], and cannot meet the development needs of modern industry. Therefore, the application of weld inspection robots has attracted extensive attention [11,12,13].

Robots play an important role in industrial manufacturing [14,15,16]. Among them, detection robots are used to replace manual detection, and they can carry various instruments and equipment for detection [17,18]. Detection robots can be divided into wheeled robots [19,20,21], crawler robots [22,23,24] and bionic robots [25,26,27,28] according to different moving modes. Wheeled robots are equipped with wheels at the bottom, which can move autonomously on a flat surface. Zhang et al. [29] proposed a wheeled welding seam detection robot, and designed a detection unit with a series-parallel flexible adaptive structure, which realized the flexible movement and effective detection of the ship wall and was used for welding seam detection to ensure safe navigation. The tank-like track structure of tracked robots allows robots to move stably on complex or uneven ground. Yang [30] proposes a crawler-type robot composed of two passive AdaGrad tracks and a connecting module suitable for ship wall features. By adjusting the posture of the moving mechanism, AdaGrad variable curvature motion can be realized, with a certain bearing capacity. A bionic robot refers to a robot with bionic principles in design and function, which is specially used to perform detection or inspection tasks. Hua et al. [31] proposed a seven-joint snake robot structure similar to sine wave, which realized the autonomous detection function of bionic snake robot. It can automatically avoid obstacles, automatically collect the data of the surrounding environment, and transmit the data to the mobile app to realize real-time monitoring. The above detection robot can not only reduce the cost of manual detection and improve the detection efficiency, but also eliminate the danger of manual detection and achieve high-quality and high-standard detection tasks.

In practical application, the mobile weld detection robot usually integrates advanced path planning and positioning technology, such as laser navigation and visual navigation, and these are used to ensure that the robot can reach the preset weld position accurately and move along it for efficient and accurate detection. Xu et al. [32] proposed a seam tracking system based on a laser vision sensor, and carried out experiments on different types of weldments. The results show that the proposed seam tracking method can achieve good tracking accuracy in most welding applications. Although laser navigation has good tracking accuracy, it requires a laser or infrared sensor on the robot, which is expensive. With the development of computer technology, machine learning is widely used in seam recognition and tracking [33,34,35]. Visual navigation uses a camera or CCD image sensor to capture the image information of the surrounding environment, and identifies the environmental characteristics through a neural network, which is associated with the actual position of the robot, thus realizing navigation and positioning [10,36]. A wall-climbing robot equipped with an industrial camera combines machine learning with traditional image processing technology, and proposes an algorithm framework for seam recognition and centerline extraction based on visual images. The actual seam recognition accuracy can exceed 90% [37]. As a core component of the machine vision domain, object detection algorithms have the advantages of high accuracy and real-time, strong scalability and easy deployment [38]. Deng et al. [39] proposed a weld feature extraction method based on the improved target detection model CenterNet. When dealing with multiple welds, this method used an independent classifier to predict the weld types to avoid false detection. Jiao et al. [40] proposed a wheel weld detection method based on the YOLOv4 algorithm. This method improves the accuracy of weld detection by optimizing the size of the anchor box and using non-maximum suppression to eliminate redundant candidate boundary boxes. Song et al. [41] put forward a new light detector light-yolo-welding for detecting weld feature points on the basis of the improved YOLOv4. Compared with other methods, this method has higher accuracy and speed, and the detection of weld feature points is more reliable and efficient.

Although the above methods can obtain some features of the weld, they cannot extract the pixel-level position information of the weld, and the rust and corrosion around the weld will affect the detection accuracy of the traditional algorithm to some extent. In order to solve the above problems, many scholars have proposed an image segmentation algorithm based on object detection [42,43]. Image segmentation decomposes an image into multiple regions or sets of pixels, and each set represents a different object or category in the image. In weld detection, the specific area of weld in the image is identified and separated by an image segmentation algorithm. Li et al. [11] put forward an intelligent inspection robot based on deep learning, which uses Mask R-CNN to segment the weld instance in weld recognition and extract the weld path with high accuracy. Yu et al. [44] used U-Net network for image segmentation, which extracted context information by combining low-level feature mapping and high-level feature mapping. The trained network can accurately detect the weld pool boundary under different welding current, welding speed, and weld pool shape. Lu et al. [45] proposed a passive vision seam tracking system for plasma arc welding based on semantic segmentation. The BiseNetV2 network and Online Hard Example Mining (OHEM) are used to improve the segmentation effect. The above method uses an image segmentation method to solve the problems of complex shape, size, and surface characteristics of the weld itself, and has strong generalization and robustness. However, the task of seam tracking detection requires high real-time accuracy. The above methods have high accuracy in seam identification, but the real-time detection effect is not ideal due to the large amount of calculation of the model adopted. Since the detection model is deployed on the robots hardware device, the size and complexity of the model need to match the computing power of the robots hardware device [46], and the model must capture the weld features quickly and accurately. YOLOv8 is an algorithm model that supports both object detection and image partitioning tasks. It has faster detection speed while maintaining relatively high detection accuracy. Therefore, this study improves the YOLOv8s-seg model to achieve a fast and high-precision segmentation of weld images. The main contributions of this study are as follows:

(1) The complex characteristics of the weld surface are simulated by a data enhancement method. This avoids the over-fitting phenomenon of the model and improves the generalization and robustness of the model.
(2) The use of a MobileNetV3 light quantization backbone network to replace the original backbone network of YOLOv8s-seg, reconstruct C2f, and prune the number of output channels of the new module C2fGhost. Finally, the EMA is added to make the improved model more suitable for fast and high-precision detection tasks.
(3) The improved model embeds the robot hardware device Jetson nano development board, and using TensorRT to accelerate model inference, the total inference time of each image is only 54 ms.

## 2. Robot Design

### 2.1. Robot Structure Design

**Figure 1:**
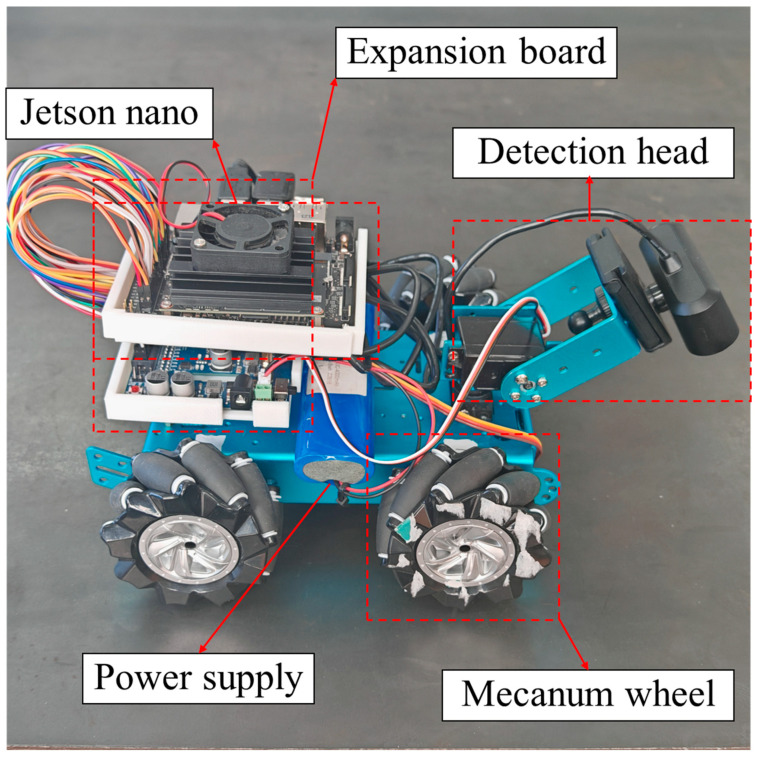
Hardware structure of the robot.

**Figure 2:**
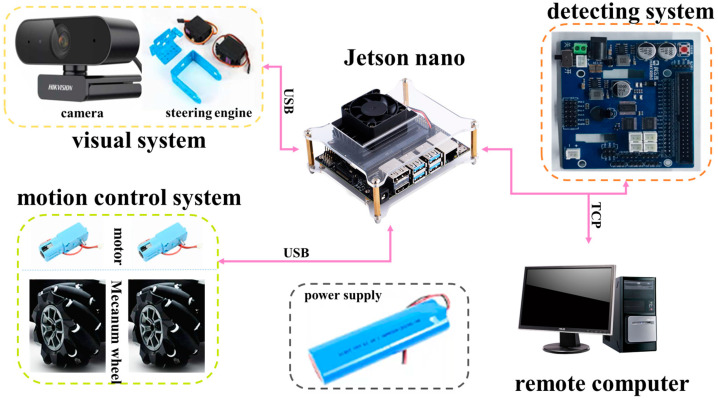
Composition of the robot system.

The whole design of the seam tracking robot consists of a detection platform, a chassis motion device, and a control system. The detection platform includes a camera with a USB port, and two servo steering gears. The camera transmits the collected image information to the control system. The servo steering gear provides the camera with two degrees of freedom in the axial direction and the circumferential direction, and realizes omni-directional scanning in the three-dimensional plane. The chassis motion device includes four motors to control four Mecanum wheels, respectively. The rotation speed of the motors is controlled by the control system, and then the rotation of the Mecanum wheels is controlled, so as to control the motion speed and direction of the car body. The control system uses the Jetson nano development board to realize visual reasoning of target weld, path fitting, and control car body movement. The structure of the robot is shown in Figure 1 and Figure 2.

### 2.2. Robots Motion Control

The weld seam segmentation model distinguishes the weld seam from the background, but the segmentation boundaries are not accurate and smooth enough. The fitting operation can optimize these boundaries by fitting, and remove irregularities and noise in the segmentation results, thereby improving the accuracy and stability of detection. This study uses the curve data coordinates fitted by the least square method for processing. By calculating the average value of the x-coordinates and y-coordinates, it is set as the center point of the weld seam, which is used to determine the position of the weld seam, which is used as the target position of the next movement of the robots.

Firstly, the coordinate system is constructed in the reasoning image with the resolution of 480 × 480, and the corresponding abscissa $x_{1}$ and ordinate $y_{1}$ of the weld center point and the corresponding abscissa $x_{2}$ and ordinate $y_{2}$ of the image center point are obtained. Secondly, the difference between the abscissa $x_{1}$ of the weld center and the abscissa $x_{2}$ of the image center, and the difference between the ordinate $y_{1}$ of the weld center and the ordinate $y_{2}$ of the image center are used:

(1) $$X=x_{1}-x_{2}$$

(2) $$Y=y_{1}-y_{2}$$

where $x_{1}$ is the abscissa of the weld center point; $x_{2}$ is the abscissa of the image center point; $y_{1}$ is the ordinate of the weld center point; $y_{2}$ is the ordinate of the weld center point; X is the abscissa difference; and Y is the ordinate difference.

When −50<X<50, it is judged to be straight, and the robot controls the motor to realize the straight command; when X<−50, it is determined that the center point of the weld is located on the left side of the robot, and the robot controls the motor to realize the left turn command; and when X>50, it is concluded that the center point of the weld is located on the right side of the robot, and the robot controls the motor to realize the right turn command, so as to realize the omni-directional tracking of the weld. In the same way, the speed of the robot is controlled by the value of *y*, and seam tracking with a controllable speed is realized. When the robots’ five consecutive reasonings $X=x_{1}$ and $Y=y_{1}$, indicating that the robots have deviated from the weld track, there is no weld image in the collected image and the system determines that the current robots have deviated from the weld track. At this time, the control system controls and detects the two-degree-of-freedom steering gear platform in the gimbal to scan the surrounding environment to find the position of the target weld. It adjusts the control system of the robots to make the robots gradually correct the motion track and realize accurate weld tracking.

### 2.3. Robot Kinematics Model

**Figure 3:**
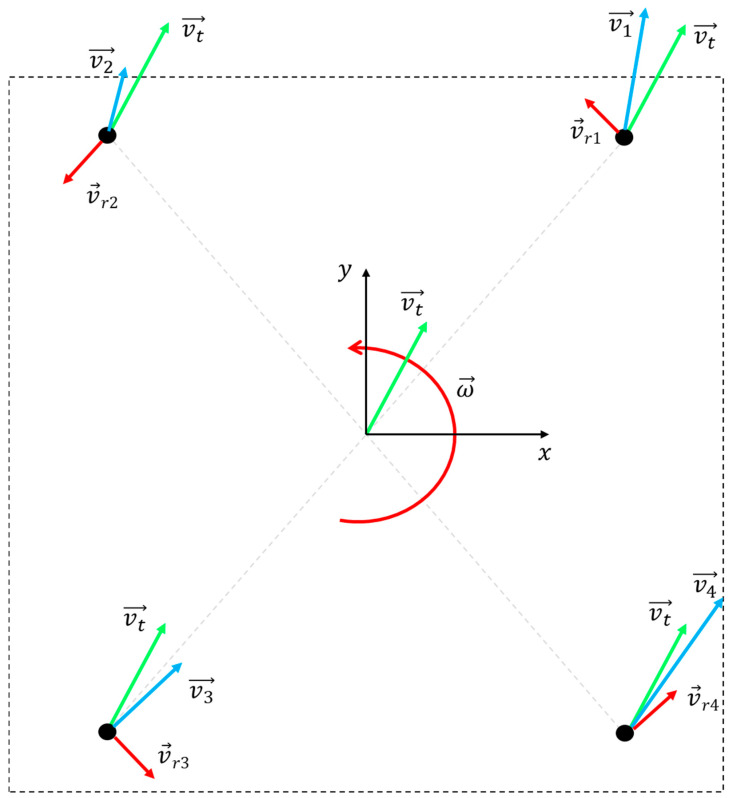
Geometric coordinate system of the robot.

Wheeled mobile robots can be divided into non-omni-directional mobile types and omni-directional mobile types according to their movement characteristics. In this paper, according to the movement characteristics of weld tracking requirements, a Mecanum wheel is adopted as the movement mode of the robot, and the special structure of a Mecanum wheel can make a translation in any direction in the plane and rotate at the same time, which meets the movement characteristics of weld tracking and allows for omni-directional mobile robots. In order to analyze the movement characteristics of a Mecanum wheel in the process of seam tracking, and further analyze the Mecanum wheel platform, we set up a coordinate system with the geometric center of the robot as the origin, as shown in Figure 3.

Weld tracking is simplified as plane motion and decomposed into independent components in three directions, i.e., X-axis translation, Y-axis translation and yaw-axis rotation. $v_{tx}$ represents the movement speed in the X-axis direction and the left–right direction, and the right direction is defined as positive. $v_{ty}$ represents the movement speed in the Y-axis direction and the front–back direction, and defines forward as positive. $\vec{\omega }$ represents the angular velocity of yaw axis rotation, and counterclockwise is defined as positive. $\vec{r}$ represents the vector from the geometric center to the wheel axis, and $\vec{v}$ is the velocity vector of the wheel axis, $\vec{v_{r}}$ is the velocity component of the wheel axis along the direction perpendicular to $\vec{r}$. Then it can be concluded that

(3) $$\vec{v}=\vec{v_{t}}+\vec{v_{r}}=\vec{v_{t}}+\vec{\omega }\times \vec{r}$$

The components of $\vec{v}$ in the X and Y axes are as follows:

(4) $$\left\{\begin{array}{c} v_{x}=v_{tx}-\omega \cdot {\vec{r}}_{y}\\ v_{y}=v_{ty}+\omega \cdot {\vec{r}}_{x} \end{array}\right.$$

**Figure 4:**
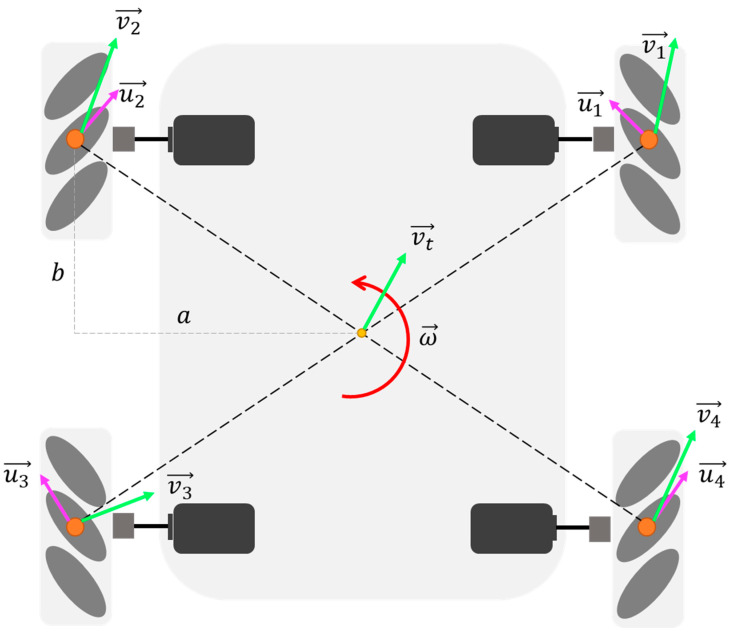
Movement exploded view of wheat wheel platform.

Similarly, the speed of the other three wheel axes can be calculated. According to the speed of the wheel axis, the speed $\vec{v_{∥}}$ along the roller direction can be decomposed as follows:

(5) $$\vec{v_{∥}}=\vec{v}\cdot \hat{u}=\left(v_{x}\hat{i}+v_{y}\hat{j}\right)\cdot \left(-\frac{1}{\sqrt{2}}\hat{i}+\frac{1}{\sqrt{2}}\hat{j}\right)=-\frac{1}{\sqrt{2}}v_{x}+\frac{1}{\sqrt{2}}v_{y}$$

where $\hat{u}$ is the unit vector along the roller direction, as shown in Figure 4.

Then the wheel speed can be obtained:

(6) $$v_{x}=\frac{\vec{v_{∥}}}{cos45{}^{\circ}}=\sqrt{2}\left(-\frac{1}{\sqrt{2}}v_{x}+\frac{1}{\sqrt{2}}v_{y}\right)=-v_{x}+v_{y}$$

According to the definitions of a and b, this can be obtained as follows:

(7) $$\left\{\begin{array}{c} v_{x}=v_{tx}+\omega b\\ v_{y}=v_{ty}-\omega a \end{array}\right.$$

According to the chassis motion state, the rotating speed of the four wheels is calculated as follows:

(8) $$\left\{\begin{array}{c} v_{\omega 1}=v_{ty}-v_{tx}+\omega \left(a+b\right)\\ v_{\omega 2}=v_{ty}+v_{tx}-\omega \left(a+b\right)\\ v_{\omega 3}=v_{ty}-v_{tx}-\omega \left(a+b\right)\\ v_{\omega 4}=v_{ty}+v_{tx}+\omega \left(a+b\right) \end{array}\right.$$

The above equations are the inverse kinematics model of the Mecanum wheel chassis, and the inverse kinematics model can be used to control the robot motion. The rotational speeds of four Mecanum wheels are calculated by the above formula, and the converted control signals are input to the driving motor controller, and then the driving wheels rotate accurately by using the control algorithm.

## 3. Weld Identification and Model Improvement

### 3.1. Construction of Weld Data Set

#### 3.1.1. Initial Weld Data Set

**Figure 5:**
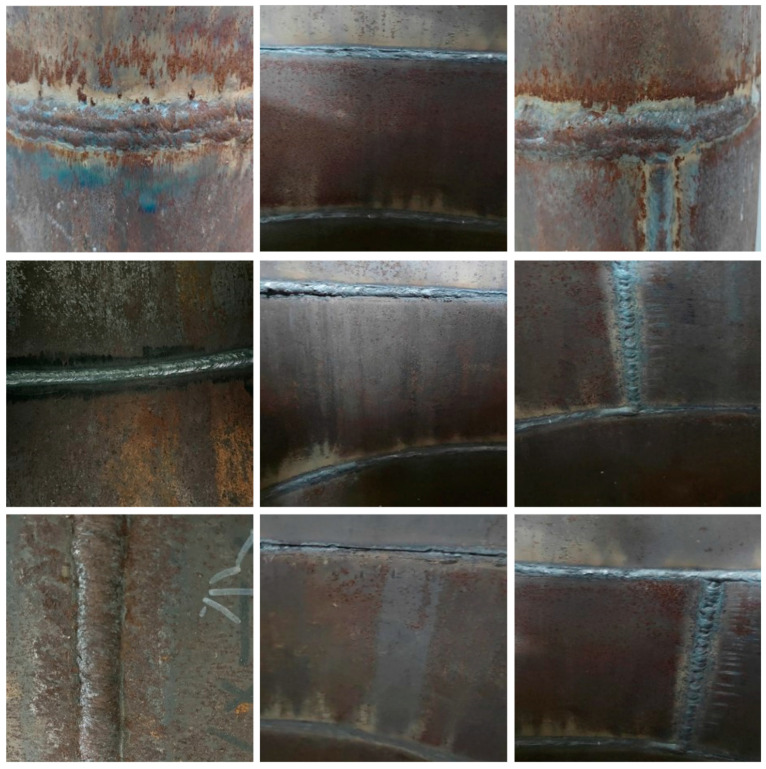
Example of original weld data set.

The initial data set used in this study included 1016 weld images, which consisted of three parts. An example of the data set is shown in Figure 5. Firstly, 300 open source data sets with the same size, environment, and background were downloaded from the Kaggle data analysis platform [47]. Secondly, 540 real weld images with different environments, backgrounds, and sizes were collected in the nondestructive testing laboratory. Finally, 176 weld images were obtained by crawling on the Internet using Python-based crawler technology. YOLOv8 is a framework of a target detection algorithm, but it also supports semantic segmentation tasks. Semantic segmentation is used to assign each pixel in an image to a specific category, so as to realize object recognition at the pixel level. Therefore, after obtaining the required data set, it is necessary to annotate the data set. In this study, the Labelme image annotation tool was used to annotate the weld images.

#### 3.1.2. Data Enhancement of Weld Image

The deep learning model needs a lot of data for training. If the training sample data are too small, the network model will be over-fitted, resulting in data distortion. In order to avoid this kind of situation, this study uses offline data enhancement and online data enhancement to enrich the data set. Before the training of the model, the existing images and labels are expanded offline. Firstly, the brightness of the weld image is adjusted to 50% and 150% of the original image, respectively. This is used to simulate the robot working in different lighting environments, and a total of 2032 weld images are obtained, which effectively prevents the over-fitting phenomenon of the model and increases the training data of the model. After data enhancement, a total of 3048 weld images were obtained. Secondly, the data set is divided into a training set and verification set according to the ratio of 7:3, and there is no data intersection between the training set and verification set.

**Figure 6:**
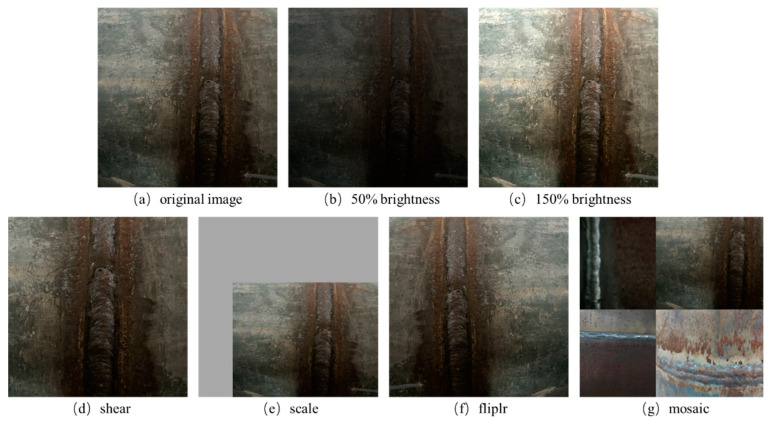
Data enhancement example.

In addition, online data enhancement is a very effective technology in model training, which can transform the input data in real time, thus increasing the generalization ability and robustness of the model. In this study, the online data enhancement strategy in YOLOv8 is used to randomly cut, scale, flip, and mosaic the image. An example of data enhancement is shown in Figure 6.

### 3.2. Weld Segmentation Model and Its Improvement

#### 3.2.1. Weld Segmentation Model

**Figure 7:**
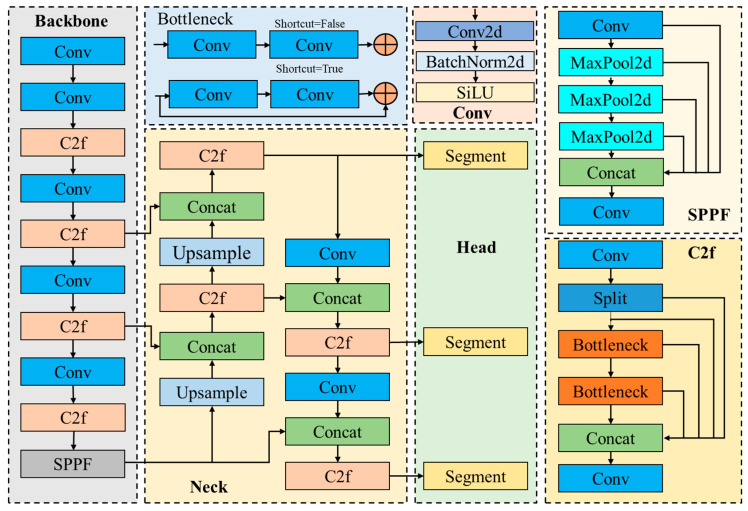
YOLOv8s-seg structure.

The detection performance of YOLOv8 is the best in the YOLO series, and it can accomplish tasks such as detection and tracking, instance segmentation, image classification, and attitude estimation. Instance segmentation can not only identify a single target in the image, but also accurately segment these targets from the image. Therefore, this study intends to use YOLOv8s-seg as the weld segmentation model. YOLOv8s-seg consists of backbone, neck, and head. backbone and neck introduce a new C2f module. There are many Bottleneck structures in the C2f module, which are connected in series, and the gradient flow of the model is enriched by more cross-layer connections. This design can effectively extract the multi-scale features in the image while ensuring a lightweight model. YOLOv8s-seg adopts the current mainstream decoupling head design. Anchor-Based is replaced by Anchor-Free, which reduces the number of prediction frames and accelerates the process of non-maximum suppression. In terms of bounding box loss, YOLOv8s-seg uses CIoU and DFL loss functions. The YOLO8s-seg structure is shown in Figure 7.

#### 3.2.2. Improve the Backbone Network

**Figure 8:**
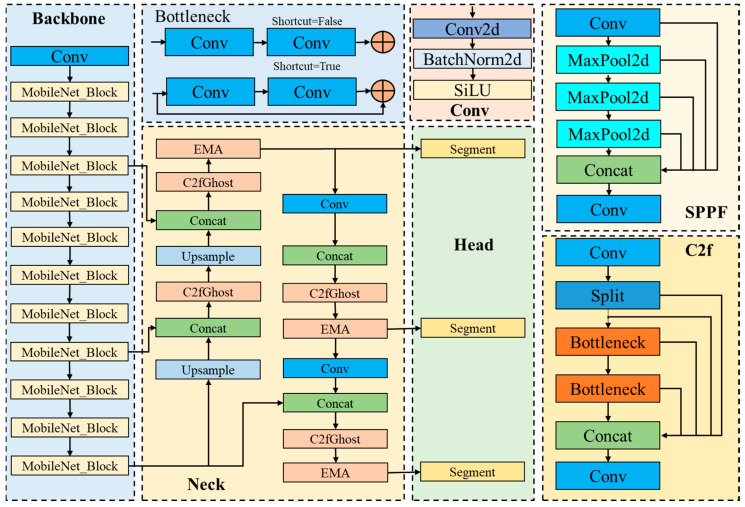
Structure diagram of the improved YOLOv8s-seg.

Although the detection performance of the YOLOv8s-seg model is superior, the welding path planning requires a model with high processing speed and accuracy. The original model has large parameters and a slow processing speed, so it needs to be improved. Therefore, in this study, the YOLOv8s-seg model is improved, and its parameters are reduced, so that it has a smaller model volume and faster processing speed. The improved model structure is shown in Figure 8.

**Figure 9:**
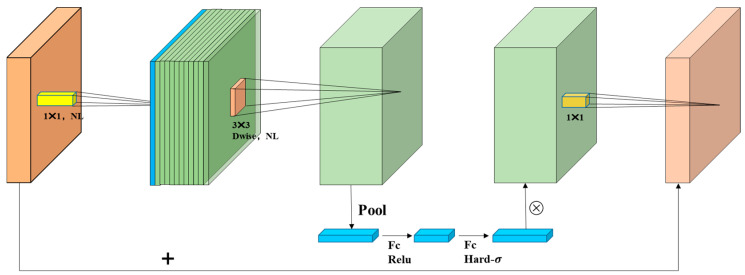
Structural diagram of MobileNetV3.

The backbone of YOLOv8s-seg adopts a CSPDarkNet-53 network, which integrates a large number of basic convolution block CBS and C2f modules. However, although this design is beneficial to improve the detection accuracy, it also inevitably brings the problems of huge parameters and a bloated model volume. A large number of weight parameters will not only increase the computational burden and limit the deployment ability in the resource-constrained environment, but also increase the storage demand and training time, which is a big challenge for the pursuit of efficient real-time applications. A lightweight improvement of the model backbone network can significantly reduce the parameters and memory occupation of the model, thus speeding up the reasoning speed. When deploying models on devices with limited resources, lightweight models can make more efficient use of CPU, GPU, or other hardware resources. In this study, the MobileNetV3 [48] network is used to replace the backbone part of the YOLOv8s-seg model. MobileNetV3 was carefully designed by Google’s research team and came out in 2019. As a deep convolutional neural network (DCNN) model optimized for mobile and embedded platforms, this architecture can ensure high-precision prediction, and at the same time greatly reduce the computational burden and memory occupation of the model, making it an ideal choice in a resource-limited environment. The structure of MobileNetV3 is shown in Figure 9. MobileNetV3 continues the efficient design of MobileNetV2 [49] in its overall architecture, and deeply integrates lightweight Depthwise Separable Convolutions as the basic building unit. This convolution solution technology greatly reduces the parameters of the model, while maintaining strong feature extraction ability. In addition, it also integrates Residual Blocks to promote gradient flow, help solve the problem of gradient disappearance in deep networks, and promote the training of deeper networks. In the improvement of its architecture, MobileNetV3 integrates a structure called Squeeze-and-Exclusion [50] (SE) in the neck part of the model. This design effectively adjusts the importance between the channels of the feature map by compressing first and then amplifying, and realizes the recalibration of the features, thus enhancing the feature expression ability of the model, and replacing the previously commonly used Swish function with H-Swish, as shown in Equations (9) and (10).

(9) $$\begin{array}{c} swish\;x=x\sigma \left(x\right) \end{array}$$

(10) $$h-\mathrm{swish}x=x*\frac{\mathrm{ReLU}6\left(x+3\right)}{6}$$

#### 3.2.3. Improve the Neck Network

**Figure 10:**
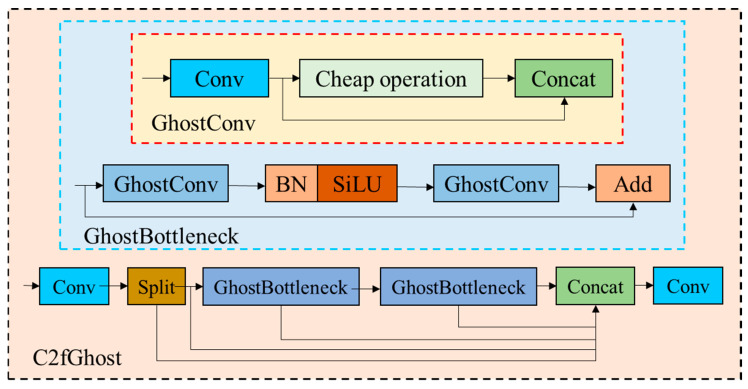
Structural diagram of C2fGhost, Ghost_Bottleneck, and GhostConv.

In order to further reduce the model parameters and realize the embedding of devices with low computational power, the C2f module in the neck part of YOLOv8s-seg model is reconstructed, and the number of output channels of C2fGhost, a new module, is trimmed. GhostBottleneck is used instead of the Bottleneck structure in the C2f module to obtain the C2fGhost module. The structures of GhostBottleneck and C2fGhost are shown in the figure. GhostBottleneck is an efficient network structure based on the GhostConv module, which is mainly composed of two stacked Ghost Convs. The first GhostConv is used as an extension layer to increase the number of channels, and the second GhostConv reduces the number of channels to match the input of the first step, so that they can add elements. By using linear transformation to generate more feature maps from existing feature maps, the required calculation amount is greatly reduced compared with relying on additional convolution layers. See Figure 10 for the structures of C2fGhost, GhostBottleneck, and GhostConv.

**Figure 11:**
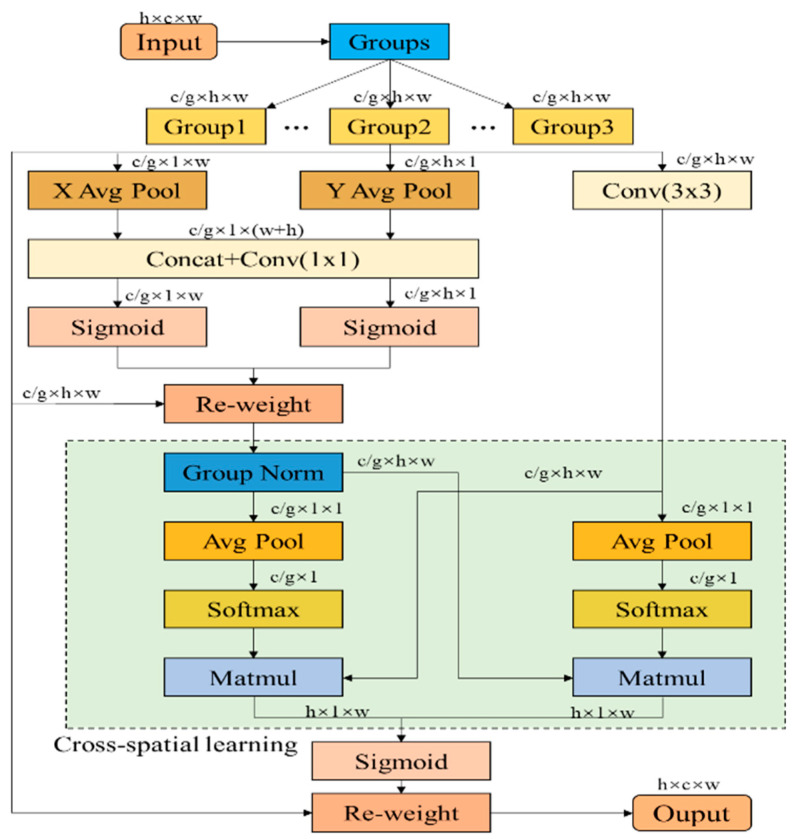
EMA structure.

The lightweight improvement of the model will inevitably lead to the loss of accuracy. In order to make up for the loss of accuracy caused by the lightweight improvement, a layer of EMA [51] (efficient multi-scale attention mechanism) is added after each detection layer in the neck part of the YOLOv8s-seg model. The maximum attention mechanism EMA abandons the process of calculating the attention diagram on the whole graph, and instead iterates a set of compact bases through an expectation maximization algorithm, and runs the attention mechanism on this set of bases, thus greatly reducing the complexity. Among them, E (efficiency) step updates the attention diagram, and M (multi-scale) step updates this set of bases. E and M are executed alternately, and are used to reconstruct the feature map after convergence. The design purpose of the EMA module is to reduce the calculation cost while ensuring that the information of each channel is preserved. It reshapes some channels into batch dimensions and groups the channel dimensions into multiple sub-features, so that the spatial semantic features are evenly distributed in each feature group. The structure of the EMA is shown in Figure 11.

### 3.3. Weld Path Fitting Method

In this project, the improved YOLOv8s-seg model is embedded in the control system of the seam tracking robot, and TensorRT is used to accelerate the reasoning of the model, so as to achieve rapid reasoning and an accurate positioning of the seam. This results in the path fitting of the robot, so as to control the robot as it inspects the weld.

#### 3.3.1. TensorRT Acceleration

TensorRT is used to accelerate the trained YOLOv8s-seg model. Firstly, the trained YOLOv8s-seg model is transformed into an onnx intermediate model, and the onnx file has high portability and operability. Secondly, the middle model onnx is optimized by an onnx optimization tool to remove redundant parameters and nodes. At the same time, the convolution layer and batch normalization layer are fused to reduce memory access operations. By reducing the complexity of the model, the calculation amount is reduced, the reasoning speed is improved, and the real-time reasoning tracking effect is realized. Finally, the optimized onnx file is converted to TensorRT, which can accelerate the inference engine file.

By using the TensorRT high-performance inference framework, the inference speed is greatly improved. This solves the problem of real-time tracking that cannot be realized due to slow inference speed.

#### 3.3.2. Least Square Method Fitting Path

TensorRT accelerates the reasoning model to obtain the reasoned segmentation mask image, and returns the position information of mask pixels in the image to obtain the two-dimensional coordinates of the weld in the original image. The parameters of the cubic polynomial are fitted by using the obtained two-dimensional coordinates through the polyfit function of numpy, and the cubic polynomial function is constructed by using the poly1d function of numpy. Then, the X-axis coordinates of the fitting curve are set in the figure according to the step size of 1, and the corresponding Y-coordinate is obtained by using a cubic polynomial through the set X-coordinate, and the path fitting is realized by approximating the fitting curve according to the obtained line chart.

The objective function of the least square cubic polynomial fitting can be expressed as follows:

(11) $$A_{1}\left(x_{1},y_{1}\right),A_{2}\left(x_{2},y_{2}\right),\ldots A_{n}\left(x_{n},y_{n}\right)$$

where A is the coefficient matrix and $x_{1},y_{1},\ldots ,x_{n}$ and $y_{n}$ represent the independent variables and dependent variables, respectively.

Therefore, the cubic polynomial obtained by the least square method can be expressed as follows:

(12) $$A_{1}\left(x_{1},y_{1}\right)A_{2}\left(x_{2},y_{2}\right)\cdots A_{n}\left(x_{n},y_{n}\right)=\frac{A_{1}\left(x_{1},y_{1}\right)+A_{2}\left(x_{2},y_{2}\right)+\cdots +A_{n}\left(x_{n},y_{n}\right)}{n+1}$$

where *n* + 1 represents the number of terms in the polynomial. The least square method is used to solve the optimal solution of $A_{1}(x_{1},y_{1})A_{2}(x_{2},y_{2})\cdots A_{n}(x_{n},y_{n})$. The seam tracking robot can obtain the path information by fitting the weld centerline to the continuous data points representing straight and curved welds, so as to better track the weld.

## 4. Experimental Results and Path Fitting Results

### 4.1. Experimental Environment

The computer operating system used in the model training stage of this study was Windows S10 Professional Edition; the CPU model was 12th Gen Intel (R) Core (TM) i5-12400f, and the GPU was NVIDIA GeForce RTX 3070. Pytorch is a framework for developing deep learning models, and its version model was 2.2.2. The compilation language was Python 3.11.8. The YOLOv8 version was Ultralytics 8.2.16. In the training stage, the mosaic data were enhanced to 1.0, the resolution of the input image was 640 × 640, the batch size was set to automatic, and epochs were set to 500 rounds. In the welding path fitting stage, the nano model used was the Jetson nano (4 GB B01).

### 4.2. Experimental Results of Weld Segmentation

**Figure 12:**
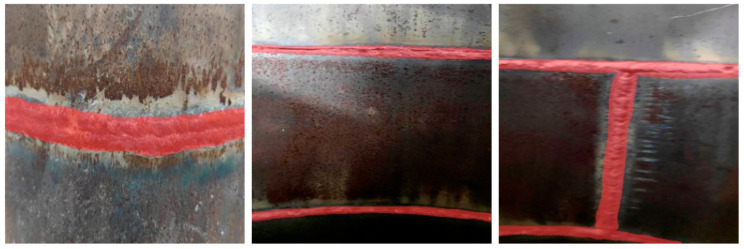
Segmentation effect of weld seam under different backgrounds.

In order to transplant the weld seam identification model to low-cost equipment, we improved the YOLOv8s-seg model. The size of the improved model is smaller, and the amount of calculation required is greatly reduced. The experimental results are shown in Table 1. The experimental results show that the size of the improved model is only 4.88 MB, which is 78.5% lower than the original model. After the lightweight improvement of the model, the improved model can still identify the weld with high accuracy, and the recognition accuracy is 97.8%, which is 0.5% higher than that of the original model. Figure 12 shows the recognition effect of the improved model on the weld in different backgrounds.

GFLOPs is an index to measure the computational complexity of the model, which is used to evaluate the computational resources required for the model to run on the hardware. The GFLOPs of the improved model is 17.7, which is about 58% lower than that of the original model. The experimental results show that the original model FPS is 51, and the improved model FPS reaches 57. We embedded the original model into the robot, and the FPS was only 1.7. The improved model embeds the robot FPS to 18.5, and the processing time of each image is only 54 ms. When nano-level devices are dealing with models, the speed of model reasoning is affected due to memory limitations or insufficient computing power, which leads to the decrease in FPS.

### 4.3. Path Fitting Results

**Figure 13:**
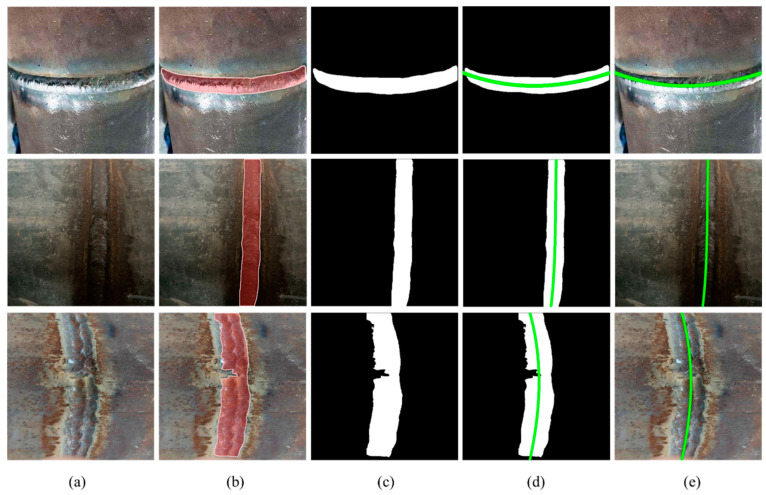
Path planning flow: (**a**) original image; (**b**) segmentation image; (**c**) image binarization; (**d**) fitted center line; and (**e**) assembly path.

In this section, a variety of complex scenes including welds are selected as experimental objects, and the performance of the weld path fitting algorithm in the weld tracking detection model is evaluated. After the improved weld segmentation model reasoning, the weld is successfully separated from the complex background, and the regional position information and the specific shape of the weld are provided. Secondly, the segmented image is transformed into a mask map by image binarization, and each pixel in the mask map is given a value to distinguish the weld from the background. Non-zero pixels in the mask map can be regarded as valid points of weld. The coordinates of these points can be extracted as the input of least square fitting. Finally, after the fitting result of the center line of the weld is obtained, the precise trajectory of the trolley can be parametrically programmed according to the fitted center line. The specific process is shown in Figure 13.

### 4.4. Weld Path Planning Experiment

**Figure 14:**
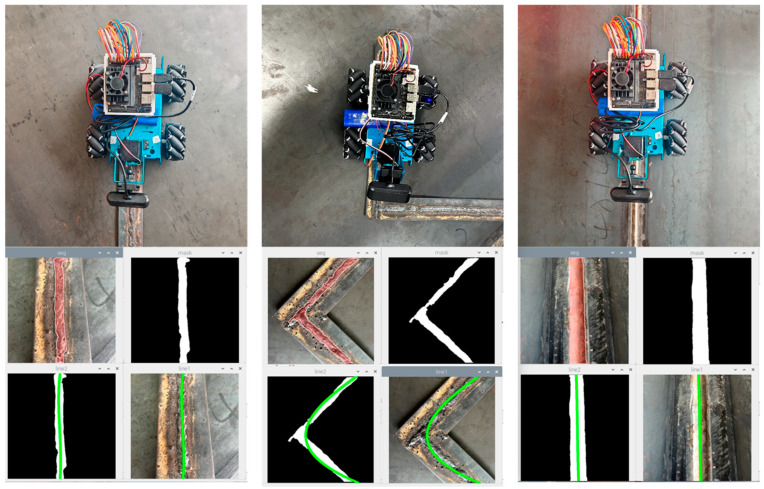
Experimental results of path planning.

In order to verify the weld path planning method proposed in this study, we put the robot in a real weld scene for experiments. The real environment consists of two steel plates with a length of 2 m, a width of 1.5 m, and a thickness of 2 cm welded along the long side, and a weld seam with a 90-degree angle welded by steel bars is added to its surface. After the robot starts running, it can view the weld data collected by the camera in real time on the computer side. In the user interface, the welding seam image can be displayed from the robot perspective, and the welding seam image processing results can be viewed to display the planned welding seam tracking path. The experimental results are shown in the figure. In the experiment, when the robot meets the weld with a 90-degree angle, it can also fit the motion route well after continuous fitting. In the weld with poor welding quality, the model can accurately identify the weld position, thus fitting the weld path. The path planning process is shown in Figure 14.

## 5. Discussion

### 5.1. Ablation Experiment

In order to verify the effectiveness of the improved YOLOv8s-seg model in the task of seam identification, four groups of experiments are designed in this section. Model 1 is the original model of YOLOv8s-seg. Model 2 replaces the backbone network of Model 1 with the lightweight network MobileNetV3. On the basis of the previous step, Model 3 reconstructs the C2f module of the neck, adds the efficient and lightweight C2fGhost module to the network, and prunes the number of output channels of C2fGhost. In Model 4, in order to make up for the precision loss caused by the lightweight model, an EMA is added after each detection layer in the neck part.

As shown in Table 2, the size of Model 2 is 12.7 MB, which is 44% lower than that of Model 1. This is because the backbone network of YOLOv8s-seg is replaced by the lightweight network MobileNetV3, which greatly reduces the calculation amount of the model and the detection accuracy is only reduced by 0.2%. Model 3 reconstructs the C2f module on the basis of Model 2 using Ghostbottleneck instead of the Bottleneck structure in the C2f module. The C2fGhost module contains the GhostConv module in its structure, which can reduce the calculation amount of the model and maintain the detection accuracy. As can be seen from the table, the detection accuracy of Model 3 is improved by 0.1%, while the size is further reduced to 4.85 MB. In Model 4, in order to make up for the precision loss caused by the lightweight model, we add the attention mechanism of EMA after each detection layer. The EMA improves the model’s ability to process features by reorganizing the channel dimension and batch dimension. The EMA module uses cross-dimensional interaction to capture the relationship at pixel level, and encodes global information in parallel branches for the recalibration of channel weights, thus enhancing the ability of feature representation. The detection accuracy of Model 4 is improved by 0.6% compared with Model 3 and 0.5% compared with the original model, and the model size is 4.88 MB. The purpose of this study is to transplant the trained lightweight and efficient model to the equipment with low calculation cost to realize the weld path planning. We verified the feasibility of the model improvement through ablation experiments, and Model 4 is the model with the best network performance.

### 5.2. Light Quantization Model Comparison Experiment

In the task of weld tracking detection, it is usually necessary to transplant the trained model to the mobile device. The mobile device has limited computing power and needs to improve the model by light quantization. In this section, the weight size, GFLOPs, mAp50, F1, and other parameters of five groups of models such as YOLOv8s-seg (N0), YOLOv8s-seg-MobileNetV3 (N1), YOLOv8s-seg-ShuffleNetV2 (N2), YOLOv8s-seg-FasterNet (N3), and Ours (N4) are compared, respectively. The experimental results are shown in Table 3.

From the experimental data, it is shown that, among the three lightweight backbone networks N1, N2, and N3, N2 and N3 models do not have obvious advantages in accuracy and model size. The model of N1 is the lightest, with the least precision loss and the smallest GFLOPs. Therefore, we further analyze the specific advantages and unique functions of MobileNetV3, and prove that it is superior to other comparison models. MobileNetV3 structurally combines the deep separable convolution of MobileNetV1 and the residual structure with the linear bottleneck of MobileNetV2, achieving a better balance between performance and efficiency. The SE attention mechanism (Squeeze-and-Excitation mechanism) is introduced to help the model learn more effective feature representation and improve the model performance. Therefore, this study made subsequent improvements on the basis of N1, and finally obtained Model N4. Compared with the original model, the segmentation accuracy of N4 is improved by 0.5%, the weight size is only 4.88 MB, GFLOPs is 17.7, and F1 is 97.2%. Compared with the original, Model N0, N4 improves the segmentation accuracy, while reducing the model size and computation, and has good detection performance.

### 5.3. Influence of Data Enhancement Strategy on Model Checking Performance

In this study, we use online data enhancement and offline data enhancement to enrich the data set. The data enhancement strategy not only improves the segmentation accuracy of the model and enhances the generalization ability and robustness of the model, but also reduces the risk of over-fitting in the model training process. This section designs a set of comparative experiments to verify the effectiveness of data enhancement. The training model with the original image data set and the training model without data enhancement are compared, and the results are shown in Table 4.

The improved model in this study is trained with a data enhancement strategy and without a data enhancement strategy. The results of weight size, GFLOPs, mAp50, and F1 are compared in Table 4. Compared with the model without a data enhancement strategy, the segmentation accuracy and F1 of the model with a data enhancement strategy are improved by 14.6% and 18.9%, respectively. This implies that the data enhancement strategy is helpful for improving the segmentation accuracy of the model, enhancing the generalization ability and robustness of the model, and also reducing the risk of over-fitting in the model training process.

### 5.4. Performance Comparison of Different Segmentation Models

In order to verify the superiority of the improved algorithm proposed in this study, this section selects the improved algorithm and the original algorithm in the latest literature and designs a comparison experiment. Since the ultimate goal of this study is to make the model embeddings in devices with low computing power, we did not select the larger models in the two-stage algorithm and the one-stage algorithm, such as Faster-RCNN [52] and YOLOv7 [53]. We selected the current mainstream one-stage algorithm and its improved model design comparison experiment, mainly including YOLOv5s-seg and YOLOv5s-segment-CA [54], YOLOv8n-seg and YOLOv8n-segment-CM [55], and YOLOv8s-seg and YOLOv8s-segment-RS [56]. Under the same conditions, the above six segmentation algorithms were used to train the weld segmentation data set. The training results are shown in Table 5.

The improved model proposed in this paper has significant advantages in weight size, mAP@0.5, GFLOPs, etc. The model size is 4.88 M, which is reduced by 78.5% compared with the original model. Compared with YOLOv5s-seg, YOLOv5s-segment-CA, YOLOv8n-seg, YOLOv8n-segment-CM, and YOLOv8s-segment-RS, it is reduced by 66.1%, 71.6%, 24.5%, 24.6%, and 84.1%, respectively; the mAP@0.5 reaches 97.8%, which is 0.5% higher than the original model. Compared with YOLOv5s-seg, YOLOv5s-segment-CA, YOLOv8n-seg, YOLOv8n-segment-CM, and YOLOv8s-segment-RS, it is improved by 2.8%, 2.2%, 7.6%, 8.3%, and 8.5%, respectively. Experimental results show that the improved model proposed in this study has better detection performance in weld segmentation. It can greatly reduce the amount of model calculation while ensuring high detection accuracy, and is more suitable for deployment in weld inspection robots.

## 6. Conclusions

In order to solve the problem of accurate weld identification and real-time tracking, a robot weld path planning system based on machine vision is proposed, and the advanced YOLOv8s-seg model is adopted as the core of the system. The model not only realizes the fine segmentation of a weld image, but could also output the accurate mask of weld, which significantly enhances the ability of weld recognition and overcomes the limitation of traditional image processing technology in accuracy. The experimental data show that the model is smaller, and is 78.5% lower than the original model, making it more suitable for transplanting low-cost equipment. In addition, the recognition accuracy of the improved model weld reaches 97.8%. In the real scene, the robot can identify the weld quickly and accurately, and plan the route accurately. The total reasoning time from image segmentation to path fitting is only 54 ms, which meets the real-time requirements of the weld tracking robot and realizes the path planning of the robot inspection weld efficiently and accurately.

In future work, we will consider adding structures such as weld defect detection and weld grinding and rust removal to the robot, upgrade the function of the intelligent detection robot, and create an all-round solution integrating weld defect detection and treatment.

## Figures and Tables

**Table 1:** Experimental results of weld segmentation.

	Weight Size	GFLOPs	mAp50	Model FPS	Robot FPS
YOLOv8s-seg	22.7	42.4	97.3%	51	1.7
Ours	4.88	17.7	97.8%	57	18.5

**Table 2:** Results of ablation experiment.

	MobilenetV3	C2fGhost	EMA	Weight Size	GFLOPs	mAp50	F1
Model 1				22.7	42.4	97.3%	97.2%
Model 2	√			12.7	29.5	97.1%	95.8%
Model 3	√	√		4.85	17.5	97.2%	97.0%
Model 4	√	√	√	4.88	17.7	97.8%	97.2%

**Table 3:** Comparative experimental results of light quantization models.

	Weight Size	GFLOPs	mAp50	F1
N0	22.7 MB	42.4	97.3%	97.2%
N1	12.7 MB	29.5	97.1%	95.8%
N2	12.8 MB	29.9	96.9%	95.7%
N3	13.0 MB	30.1	96.7%	96.2%
N4	4.88 MB	17.7	97.8%	97.2%

**Table 4:** Experimental results of data enhancement.

	Weight Size	GFLOPs	mAp50	F1
With data enhancement	4.88 MB	17.7	97.5%	97.2%
Without data enhancement	4.83 MB	17.7	82.9%	78.3%

**Table 5:** Segmentation results of different models.

	Weight Size	maP50	GFLOPs
YOLOv5s-seg	14.4 MB	95.0%	25.7
YOLOv5s-seg-CA	17.2 MB	95.6%	28.6
YOLOv8n-seg	6.47 MB	90.2%	12
YOLOv8n-seg-CM	6.48 MB	89.5%	12.1
YOLOv8s-seg	22.7 MB	97.3%	42.4
YOLOv8s-seg-RS	30.7 MB	89.3%	54.6
Ours	4.88 MB	97.8%	17.7

## Data Availability

All data included in this study are available upon request by contacting the corresponding author.
